# The CFTR Mutation c.3453G > C (D1152H) Confers an Anion Selectivity Defect in Primary Airway Tissue that Can be Rescued by Ivacaftor

**DOI:** 10.3390/jpm10020040

**Published:** 2020-05-13

**Authors:** Onofrio Laselva, Theo J. Moraes, Gengming He, Claire Bartlett, Ida Szàrics, Hong Ouyang, Tarini N. A. Gunawardena, Lisa Strug, Christine E. Bear, Tanja Gonska

**Affiliations:** 1 Programme in Molecular Medicine, Research Institute, Hospital for Sick Children, Toronto, ON M5G 8X4, Canada; onofriolaselva@gmail.com (O.L.); ida.szarics@mail.utoronto.ca (I.S.); bear@sickkids.ca (C.E.B.); 2 Department of Physiology, University of Toronto, Toronto, ON M5G 8X4, Canada; 3 Programme in Translational Medicine, Research Institute, Hospital for Sick Children, 555 University Avenue, room 8415, Toronto, ON M5G 8X4, Canada; theo.moraes@sickkids.ca (T.J.M.); claire.bartlett@sickkids.ca (C.B.); hong.ouyang@sickkids.ca (H.O.); tarini.gunawardena@sickkids.ca (T.N.A.G.); 4 Department of Paediatrics, University of Toronto, Toronto, ON M5G 8X4, Canada; 5 Programme in Genetics and Genome Biology, Research Institute, Hospital for Sick Children, Toronto, ON M5G 8X4, Canada; gengming.he@sickkids.ca (G.H.); lisa.strug@sickkids.ca (L.S.); 6 The Centre for Applied Genomics, Hospital for Sick Children, Toronto, ON M5G 8X4, Canada; 7 Department of Statistical Sciences and Division of Biostatistics, University of Toronto, Toronto, ON M5G 8X4, Canada; 8 Department of Biochemistry, University of Toronto, Toronto, ON M5G 8X4, Canada

**Keywords:** cystic fibrosis, personalized medicine, D1152H, CFTR, VX-770, rare mutation

## Abstract

The Cystic Fibrosis Transmembrane Conductance Regulator (CFTR) gene variant, c.3453G > C (D1152H), is associated with mild Cystic Fibrosis (CF) disease, though there is considerable clinical variability ranging from no detectable symptoms to lung disease with early acquisition of *Pseudomonas aeruginosa*. The approval extension of ivacaftor, the first CFTR modulator drug approved, to include D1152H was based on a positive drug response of defective CFTR-D1152H chloride channel function when expressed in FRT cells. Functional analyses of primary human nasal epithelial cells (HNE) from an individual homozygous for D1152H now revealed that while CFTR-D1152H demonstrated normal, wild-type level chloride conductance, its bicarbonate-selective conductance was impaired. Treatment with ivacaftor increased this bicarbonate-selective conductance. Extensive genetic, protein and functional analysis of the nasal cells of this D1152H/D1152H patient revealed a 90% reduction of CFTR transcripts due to the homozygous presence of the 5T polymorphism in the poly-T tract forming a complex allele with D1152H. Thus, we confirm previous observation in patient-derived tissue that 10% normal CFTR transcripts confer normal, wild-type level chloride channel activity. Together, this study highlights the benefit of patient-derived tissues to study the functional expression and pharmacological modulation of CF-causing mutations, in order to understand pathogenesis and therapeutic responses.

## 1. Introduction

Cystic fibrosis is a recessive genetic disease caused by mutations in the Cystic Fibrosis Transmembrane Regulator *(CFTR)* gene, which codes for an anion channel involved in the conduction of both, chloride and bicarbonate ions [1,2,3]. Small molecule interventions targeting the basic defect(s) caused by CFTR mutations have transformed the therapeutic landscape for Cystic Fibrosis [4,5]. One is ivacaftor (also known as KALYDECO™, VX-770) [6], which potentiates the chloride channel activity of wild-type CFTR and several rare mutant CFTR proteins [7]. The drug efficacy of ivacaftor was first demonstrated for c.1652G > A (pGly551Asp, G551D) expressed in heterologous expression systems [6,8,9]. These experiments were then validated in patient derived primary bronchial tissues before advancing into clinical trial. The subsequent success of ivacaftor in rescuing lung function and health for individuals with G551D in phase 3 clinical trials let to its approval by the US Food and Drug Administration (FDA) [10]. Interestingly, label extension of ivacaftor for other rare CFTR mutation was basis on testing their drug response when expressed in heterologous cell models [11]. For example, the use of ivacaftor for the treatment of individuals bearing the rare mutation, c.3454G > C (p.Asp1152His, D1152H), was approved on the basis of a positive chloride channel response to ivacaftor by this mutant when expressed in Fischer Rat Thyroid (FRT) cells [7]. 

Individuals who have the D1152H mutation on at least one allele, typically exhibit mild disease although there is considerable clinical variability (Table 1) (https://www.cftr2.org). The clinical phenotype can range from no detectable symptoms to lung disease with early acquisition of *Pseudomonas aeruginosa*, a classic CF pathogen responsible for lung infection [12,13,14,15]. Previous studies of the mutant D1152H protein expressed in FRT cells showed that its biosynthesis as a complex glycosylated protein was normal and it exhibited a minor reduction of its channel activity of 78 per cent of Wt CFTR function, a decrease that is not expected to lead to physiological abnormalities (https://www.cftr2.org). Hence, studies of the D1152H mutant in FRT cells fail to define the primary defect underlying disease and thus the rationale for treatment with ivacaftor remains unclear. 

In this study, we collected nasal epithelial tissue for DNA and RNA sequencing and prepared well differentiated primary nasal epithelial cell cultures from an individual homozygous for D1152H to evaluate the expression and function of this mutant in a relevant tissue context. 

## 2. Materials and Methods

### 2.1. Cell Culture and Transfection

Human embryonic kidney 293 GripTite™ cells (HEK293) (a gift from Dr. Daniela Rotin, Hospital for Sick Children, Toronto, ON, Canada) were maintained in DMEM (Wisent, St-Bruno, QC, Canada) supplemented with non-essential amino acids (Life Technologies, Waltham, MA, USA) and 10% fetal bovine serum (FBS; Wisent) at 37 °C and processed with 5% CO_2_ as previously described [16,17]. Transient transfections were performed using PolyFect Transfection Reagent (Qiagen, Hilden, Germany), according to the manufacturer’s protocol, as previously described [18,19].

The nasal brushing and subsequent cell culture was performed as previously described [20,21,22]. Briefly, the brushing was performed by a research nurse (JA) and epithelial cells from the inferior turbinate were obtained. Cells were dissociated gently from the brush and cultured in warm expansion media (PneumaCult Ex, StemCell Tech., Vancouver, BC, Canada) containing 5 µM Y27632 (Selleckchem, Cedarlane, Burlington, ON, Canada) and antibiotics. Cells were then expanded in submerged culture for 1 cell passage (P1) and subsequently seeded on collagen coated Transwell inserts (P2-3) (6.5 mm diameter, 0.4 µm pore size, Corning, Tewksbury, MA, USA) at a density of 10e5 cells per insert. Once confluent, the media was changed to air liquid interface (ALI) with basal differentiation media (PneumaCult^TM^ ALI, StemCell Tech.). By 2 to 3 weeks, cells were well differentiated with a ciliated phenotype [20,21,23].

### 2.2. Immunoblotting 

HEK293 and nasal cells were lysed in modified radioimmunoprecipitation assay (RIPA) buffer (50 mM Tris-HCl, 150 mM NaCl, 1 mM EDTA, pH 7.4, 0.2% SDS, and 0.1% Triton X-100) containing a protease inhibitor cocktail (Roche, Mannheim, Germany) for 10 minutes. Soluble fractions were analyzed by SDS-PAGE on 6% tris-glycine gels (Life Technologies, Carlsbad, CA, USA). After electrophoresis, proteins were transferred to nitrocellulose membranes (Bio-Rad, Hercules, CA, USA) and incubated in 5% milk. In HEK293 cells, CFTR bands were detected with human CFTR-specific murine mAb 596 with 1:5000 dilution and in nasal cultures with 1:500 dilution. The blots were developed with ECL (Amersham) using the Li-Cor Odyssey Fc (LI-COR Biosciences, Lincoln, NE, USA) in a linear range of exposure (2–45 min) [24,25]. Relative levels of CFTR proteins were quantitated by densitometry of immunoblots using ImageStudioLite (LI-COR Biosciences, Lincoln, Nebraska, USA) [26,27].

To evaluate protein glycosylation status, lysates were treated with 500U of endoglycosidase H (New England Biolabs, Ipswich, MA, USA) according to the manufacturer’s protocol. 

### 2.3. CFTR Channel Function in HEK293 Cells

HEK293 cells were seeded in 96-well plates (black, flat bottom). After 24 h the cells were transfected with either WT-, D1152H- or D1152H/exon10del-CFTR. Functional studies were performed 48 h post-transfection. Cells were loaded with blue membrane potential dye for dissolved in chloride free buffer for 45 minute at 37 °C. The plate was then read in a fluorescence plate reader (SpectraMax i3; Molecular Devices, San Jose, CA, USA) at 37 °C, and after reading the baseline fluorescence (excitation: 530 nm, emission: 560 nm) for 5 min, CFTR was stimulated using the forskolin (10 µM) +/− ivacaftor (VX-770,1 µM). CFTR-mediated depolarization of the plasma membrane was detected as an increase in fluorescence. Then, CFTR inhibitor (CFTRinh-172, 10 µM) was added to deactivate CFTR. The peak changes in fluorescence to CFTR agonists were normalized relative to fluorescence immediately before agonist (forskolin) addition [24,28,29].

### 2.4. Ussing Chamber Studies of Primary Nasal Epithelial Cells

Primary nasal cells were grown on transwells and studied in a non-perfused Ussing chamber (Physiologic Instruments, San Diego, CA, USA). The buffer solutions were as follows: (in mM) Krebs bicarbonate: 126 NaCl, 0.38 KH_2_PO_4_, 2.13 K_2_HPO_4_, 1 MgCl_2_, 1 CaCl_2_, 24 NaHCO_3_, 10 glucose. Zero chloride buffer: 116.2 Na gluconate, 2.4 KH_2_PO_4_, 1.24 K_2_HPO_4_, 1 MgSO_4_, 1 Ca gluconate, 25 NaHCO_3_, 10 glucose. zero bicarbonate buffer: 145 NaCl, 3.3 K_2_HPO_4_, 10 HEPES, 1.2 MgCl_2_, 1.2 CaCl_2_, 10 glucose, pH adjusted with acetic acid. Bath solutions were maintained at pH 7.4 and 37 °C and continuously gassed with a 5% CO_2_/95% O_2_ mixture or air in the case of bicarbonate free buffer. The transepithelial potential (Vte) was recorded in open-circuit mode and the baseline resistance (Rte) was measured following repeated, brief short-circuit current pulses (1 µA every 30 s). The results are presented as equivalent transepithelial current (Ieq), which was calculated using Ohm’s law. CFTR function was determined after inhibition of the epithelial sodium channel (ENaC) with amiloride (30 µM, Spectrum Chemical, Gardena, CA, USA) and following cAMP activation with forskolin (10 µM, Sigma-Aldrich, St. Louis, MO, USA). CFTR activity was confirmed as Ieq difference following CFTR inhibition with CFTRInh_-172_ (10 µM, EMD Millipore Corp, Burlington, MA, USA) [22].

### 2.5. RNA Sequencing 

Details of RNA extraction, quality control and data processing are described in a recent publication [23]. In brief, nasal epithelial cells are obtained by a cytology brush and are immediately inserted into RNA Later RNA stabilization reagent. The RNeasy Tissue RNA extraction kit (ThermoFisher, Waltham, MA, USA) are used for RNA extraction following the manufacturer’s instruction. The extracted RNA has a RNA integrity number (RIN) equals to 10 (measured by Agilent Bioanalyzer 2100 RNA Nano chip). RNA sequencing libraries were generated using NEBNext Ultra II Directional RNA library prep kit. Libraries were sequenced using the Illumina HiSeq 2500 platform (Illumina, San Diego, CA, USA) in high output mode, generating about 30 million paired-end reads (2x126 bp). The quality of raw reads was assessed by FastQC [30]. The low quality read ends (Phred score < 20) and reads shorter than 40 base pairs were removed by Trim Galore! [31]. The trimmed reads were aligned to the hg19 human genome reference using STAR [32]. A Sashimi plot was used for visualization and was generated by the Integrative Genomics Viewer (IGV) [33,34] using the aligned reads.

The T-tract was assessed by the Genome Diagnostics laboratory at the Hospital for Sick Children using the xTAG^R^ CFTR Luminex assay. 

## 3. Results

### 3.1. Overexpression in a Heterologous System Shows Reduced Function of D1152H-CFTR

In order to recapitulate earlier reports of functional alteration of the D1152H-CFTR mutant [7], we used human embryonic kidney (HEK) cells to overexpress D1152H for functional studies. We employed the FLIPR assay, which is an established fluorescence-based technique measuring membrane potential change in response to different agonists or inhibitors. In D1152H-CFTR expressing cells CFTR function measured as forskolin-induced and CFTR_Inh-172_—sensitive membrane potential change was reduced to about 70% of Wt-CFTR chloride channel activation (Figure S1C,D). 

The Western blot analysis showed no difference in protein abundance (normal band B) or maturation (normal band C) compared to the Wt-CFTR protein (Figure S1A,B). As previously demonstrated [7], these data suggest that the D1152H-mutant does not showed protein processing defect. 

### 3.2. RNA and DNA Sequencing of Nasal Tissue Derived from Patients Homozygous for D1152H Revealed a Splicing Defect Attributed to the 5T-polyT Tract Polymorphism Present on Both Alleles

To investigate the molecular defect of D1152H mutation, we used nasal epithelial cells from a CF patient homozygous for D1152H. Interestingly, while the heterologous system showed only 70% of Wt-chloride channel activity, this patient exhibited normal lung function (FEV1 97.8%, Figure S2) and pancreatic sufficiency. Moreover, this patient had experienced lung infections with *Pseudomonas aeruginosa*, a classic CF pathogen (Table 2). Together, these findings suggest that this individual exhibits a mild course of cystic fibrosis. 

**Figure 1:**
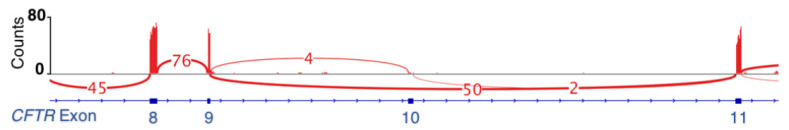
The sashimi plot for RNA sequencing reads aligned to CFTR exon 8 through exon 11. Read counts represent the number of reads aligned to each base in the exon. Bridges connecting exon pairs show the number of reads that span sequence in the two neighbouring exons (i.e., aligned to splice junctions). For the 56 reads aligned to the splice junctions spanning exons 9, 10 and 11, 50 (89.3%) skipped exon 10 and show direct connections between exons 9 and 11.

We then asked if this mild disease was associated with altered CFTR expression. Fresh nasal epithelial cells were obtained from this patient and processed for RNA and DNA sequencing. Analysis of the RNAseq data showed that 50 out of 56 splice junction reads directly aligned with *CFTR* exon 9 and exon 11 with exon 10 skipped, visualized in an IGV-Sashimi plot (Figure 1). This analysis indicated a loss of exon 10 in ~90% of the CFTR splice products in the nasal cells of our patient (Figure 1). This finding is consistent with the impact of an intron 9 5T polymorphism, as seen for other CF complex alleles [35,36]. We confirmed homozygosity of the 5T poly-T tract polymorphism through CFTR genotyping, thus establishing presence of D1152H with 5T on both alleles for our patient.

### 3.3. Complex Mutant CFTR (D1152H-exon10del) Resulted in Reduced CFTR Protein in Cells

**Figure 2:**
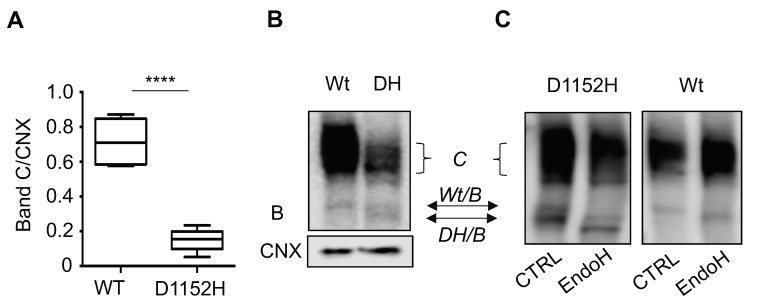
Nasal epithelial cells derived from one patient homozygous for the D1152H mutation exhibit protein processing defects. (**A**) Bar graphs represent the mean (±SD) of the CFTR protein expression in nasal cells from healthy control (WT) compared to our D1152H/D1152H patient as ratio band C to calnexin C/CNX from 5 independent experiments. (**B**) Representative immunoblot from nasal cells generated from a healthy control (WT) and the D1152H/D1152H patient. C: mature, complex-glycosylated CFTR; B: immature, core-glycosylated CFTR. (**C**) Immunoblot shows the sensitivity of band B of the D1152H-CFTR in nasal epithelial cultures to endoglycosidase H (EndoH). (**** *p* < 0.0001).

Immunoblotting of the compound mutant CFTR (D1152H-exon10deletion) in patient derived nasal epithelial cells revealed a different protein migration pattern compared to the pattern obtained in the HEK-293 overexpression system (Figure 2A and Figure S1A). The nasal cells from the individual with the complex mutant showed a lower abundance of the mature form (band C) of the CFTR protein when compared to Wt-CFTR, which was in the range of approximately 10–20% of Wt-CFTR protein (Figure 2A,B). This finding matched the RNAseq data showing 10% of normal length CFTR transcripts. The immature form of the D1152H-exon10del CFTR protein (band B) was split into two smaller bands, which were core-glycosylated (EndoH-sensitive) (Figure 2C). We suggest on the basis of our findings in the HEK-293 system (Figure S1A), that the smaller and relatively dominant band B, corresponds to the form lacking amino acids 404–464 in the NBD1domain of the CFTR protein due to exon 10 deletion.

Furthermore, as expected, the recombinant protein D1152H/exon10del, is not active as a cyclic AMP-regulated chloride channel when expressed in HEK-293 cells, since the membrane localized form of the protein is significantly reduced and since it is lacking a functionally significant region in NBD1 (the amino acid sequence 404–464 includes the Walker A lysine, which is important for ATP binding and hydrolysis) (Figure S1E,F). Further, the CFTR modulators, ivacaftor (VX-770) and tezacaftor (VX-661) fail to rescue the function of this complex mutant in HEK-293 cells (Figure S1E,F). This latter finding was not surprising as in this system there is insufficient mature full -length protein amenable to tezacaftor or ivacaftor rescue.

### 3.4. Nasal Cell Cultures Derived from Patient Homozygous for D1152H Exhibit Altered Bicarbonate Conductance 

Given that the nasal cells from the affected subject contained reduced amounts of the full-length protein, we were prompted to determine if this was sufficient to support normal CFTR channel function. 

**Figure 3:**
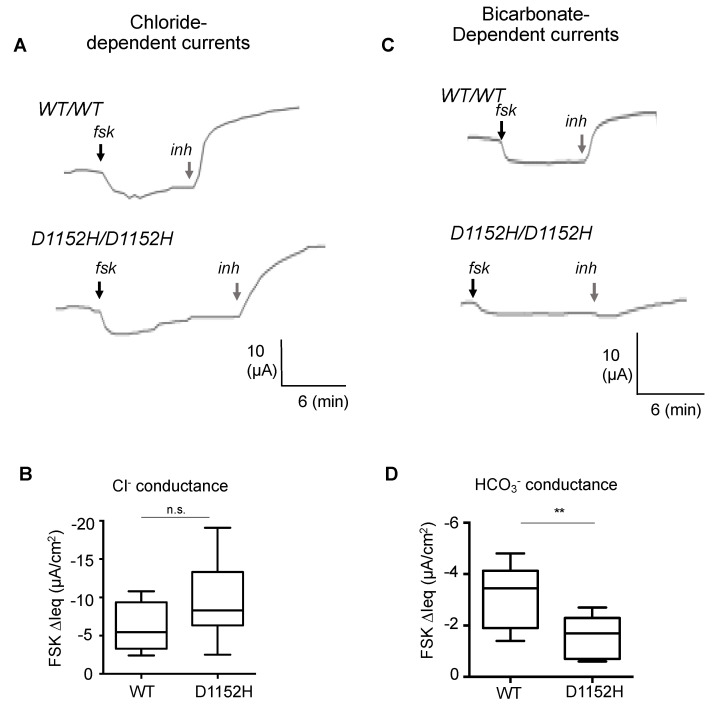
Nasal epithelial cultures derived from a patient homozygous for the D1152H mutation exhibit a bicarbonate conductance defect. Representative current tracings from Ussing chamber studies in (**A**) Bicarbonate-free buffer to study chloride conductance and (**C**) Chloride-free buffer to study bicarbonate conductance of the CFTR channel in nasal cell cultures from CF patient carrying D1152H/D1152H compared to a healthy control (WT/WT). (**B**,**D**) Bar graph showing mean (±SD) forskolin (10 µM) activated ∆Ieq for nasal cultures of healthy controls and D1152H/D1152H CF patient (*n* = 6–9 replicates). (** *p* < 0.01).

**Figure 4:**
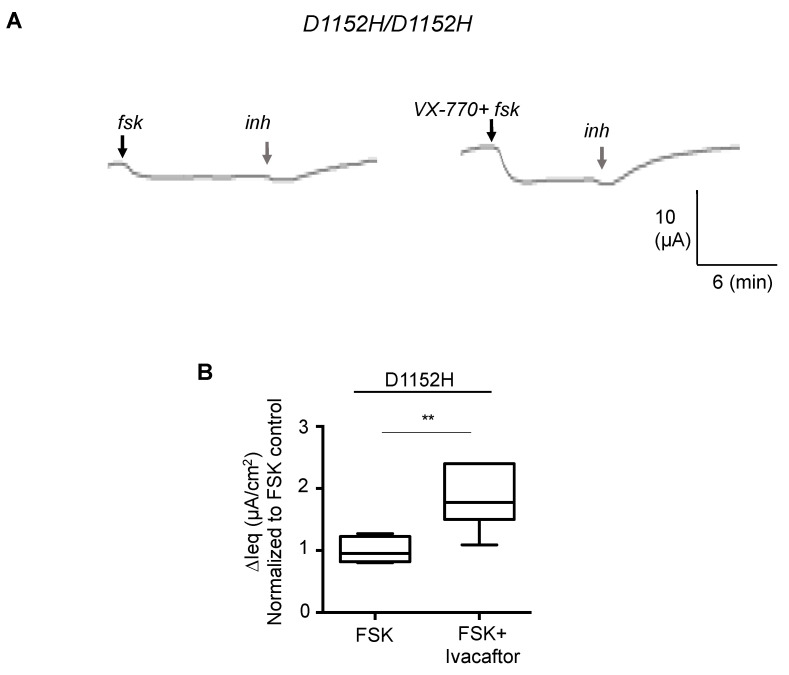
The bicarbonate conductance defect is rescued by ivacaftor. (**A**) Representative current tracings from Ussing chamber studies in Chloride-free buffer to study bicarbonate conductance of the CFTR channel in nasal cell cultures from CF patient carrying D1152H/D1152H. (**B**) Bar graph showing the fold increased forskolin (10 µM) +/− VX-770 (1 µM) activated ∆Ieq compared to forskolin (10 µM) control (*n* = 7 replicates). (** *p* < 0.01)

As shown in Figure 3A,B, we performed Ussing chamber studies using chloride and bicarbonate substituted perfusion baths to separately evaluate the chloride and bicarbonate conductance properties of the CFTR-D1152H in the patient’s nasal cells. Interestingly, we found no difference in the chloride conductance when compared to non-CF nasal cell cultures, but a significant reduction in the bicarbonate conduction. (Figure 3C,D). Treatment with ivacaftor increased the bicarbonate conductance by two fold (Figure 4).

Altogether, our studies suggest that studies of patient derived tissue are essential to understand the individual molecular and functional CFTR defect, particularly with rare CFTR mutations. RNA and DNA sequencing revealed a splicing defect that resulted in low abundance of properly processed, full-length protein. While 10% of full-length CFTR transcript bearing D1152H was sufficient to confer normal chloride conductance, electrophysiological studies revealed a defect in bicarbonate conductance rescuable with ivacaftor treatment. Since the maintenance of a relatively alkaline pH is important for innate host defense, this defect may have accounted for the *Pseudomonas aeruginosa* infection observed in this individual. 

## 4. Discussion 

In this study, we showcased how comprehensive studies using patient derived nasal cells led to the discovery of complex alleles in an individual homozygous for D1152H and 5T, and also provided further insight into the molecular defect of the missense mutation, D1152H. In contrast to previous studies D1152H does not alter transepithelial CFTR mediated chloride, but bicarbonate conductance. 

Normal CFTR-mediated chloride secretion may account for absence of typical CF clinical phenotypes, such as altered sweat chloride concentrations and compromised lung function in this D1152H homozygote individual. Given the lack of pancreatic disease, the residual bicarbonate conductance mediated by D1152H-CFTR in this individual is likely sufficient to spare disease pathogenesis in this organ. However, the sporadic infection with *Pseudomonas aeruginosa* in this patient argues that the reduced bicarbonate conductance mediated by D1152H renders the airways susceptible to colonization, as previously suggested [37,38]. 

As previously discussed, analysis of the RNAseq data obtained from the nasal cells of our patient, revealed a loss of exon 10 in ~90% of the CFTR splice products due to the poly-T tract 5T variant, present on both alleles together with D1152H. As the loss of exon 10 abrogates protein expression and function as confirmed in studies of the recombinant exon 10 deletion mutant (Figure S1), our findings that the patient’s nasal cultures exhibited normal chloride conductance support the concept that only 10% of the full length CFTR transcript is sufficient to confer Wt CFTR function. In fact, Ramalho et al. suggested that 5% of full-length CFTR mRNA is enough to ameliorate the severity of CF disease on the basis of studies of nasal cells acquired from 5 CF patients bearing a variant that confers aberrant splicing: 3272-26A and a mild clinical phenotype [39]. 

Previous studies of the impact of the missense mutation D1152H on CFTR chloride conductance employed multiple expression systems and reported variable results [7,38,40]. As already mentioned earlier, studies of D1152H expressed in FRT cells and reported in the CFTR2 portal (https://www.cftr2.org), showed that D1152H caused a minor reduction in its channel activation to 78% Wt chloride channel activity. In *Xenopus laevis* oocyte expression studies, D1152H expression conferred severely defective currents, close to 12% of the Wt-CFTR chloride conductance [40]. In HEK-293 cells, LaRusch et al. showed that the CFTR chloride channel activation conferred by D1152H was essentially the same as Wt-CFTR [38]. These discordant findings reflect the importance of the cellular context and the protein interactome in modulating CFTR function. Our studies of D1152H-CFTR protein in the apical membrane of primary nasal epithelial cultures, showing normal CFTR chloride channel function may reflect the phenotypic consequences of this mutation in the most relevant context to date. 

Similar to the studies by LaRusch et al. in the HEK-293 expression system [38], we observed a modest decrease in bicarbonate conductance for D1152H in primary nasal epithelial cultures. The specific impact of D1152H on bicarbonate conductance rather than chloride conductance may reflect the chemical properties of the bicarbonate ion and/or signaling pathways that regulate anion permeation through the pore [24,41]. The bicarbonate ion is larger than chloride ion and according to molecular dynamic simulations, the D1152H substitution may introduce a barrier to the internal vestibule that specifically affects bicarbonate ion access. The relative permeability of the pore to bicarbonate ions is also dependent on intracellular chloride ion concentrations and WNK1-SPAK signaling [38,42,43]. It remains possible that the D1152H substitution alters the interaction of CFTR with this signaling pathway and this concept will be investigated in future studies. 

According to LaRusch et al., the modest decrease in bicarbonate conductance through D1152H, may lead to pathology in tissues and organs that depend on this CFTR function, including the pancreatic duct, nasal sinuses and the male reproductive tract. In fact, LaRusch reported that this mutation was found in non-CF patients with pancreatitis who also had an increased risk of rhinosinusitis and male infertility [38]. Interestingly, our patient who had both the 5T polymorphism and D1152H on both alleles and a bicarbonate conductance defect, did not have pancreatitis; pancreatitis is not uniformly reported in patients bearing D1152H [12,15]. In a large study using the French CF registry 2/3 of the 42 CF patients who expressed D1152H at least on one allele. showed signs of lung disease and 1/3 were pancreatic insufficient as adults. In the North American Pancreatitis Study 2 registry, which is the largest collection of adult patients with pancreatitis in North America, the frequency of D1152H among patients with pancreatitis was only 0.4% [38]. There are single reports noting a higher D1152H allele frequency in patients with CBAVD [44]. In our own cohort of patients with a CFTR-related disease, D1152H was not identified at a higher frequency in patients with CBAVD or sinopulmonary disease [45,46].

Importantly, we also show in relevant, patient derived tissues that ivacaftor treatment does exert a rescue effect, but interestingly not on the phenotype for which it was approved. Ivacaftor was approved for treatment of individuals having D1152H based on data showing this mutation had a defect in chloride conductance. However, we demonstrated that, in our patient, this mutation does not affect chloride conductance, rather it exhibits reduced bicarbonate conductance, which was increased with ivacaftor treatment. The mechanism by which ivacaftor potentiates bicarbonate conduction has yet to be fully understood, although studies by Moran and colleagues suggest it can change the selectivity of the CFTR channel pore [47]. It may be that the therapeutic effect of ivacaftor on other rare mutations is dependent on its specific potentiation of bicarbonate conductance as well and this hypothesis will be addressed in our future work.

To summarize, these studies emphasize the need to investigate expression and function of mutant CFTR in its native epithelial context in order to understand the molecular basis for disease and pharmacological responses for mutations associated with atypical Cystic Fibrosis.

## Figures and Tables

**Table 1:** Clinical information on a total of 556 patients from the CFTR2 database.

	No. of pts	Average Age (Years)	Sweat Cl (mmol/L)	Lung Function (Age 10–20) %	%PI	PA Rate (%)
D1152H/D1152H	15	16	38	na	0	15
D115H/F508del	358	29	44	70–122	27	36
D1152H/ any PI gene variant	495	29		75–123	24	36

PI—pancreas insufficiency, PA = Pseudomonas aeruginosa.

**Table 2:** Clinical data of the patient homozygous for D1152H/5T.

Age	21 Years	37 Years
Date of Diagnosis	16 years	
Sweat chloride (mmol/L)	34 and 33	
Pancreas status	sufficient	
BMI kg/m^2^	19.7	22.1
FEV1 (%pred)	92.6	97.8
FVC (%pred)	88.7	92.6
Pseudomonas aerug.	Grew once at 21years of age	

## Supplementary Materials

The following are available online at https://www.mdpi.com/2075-4426/10/2/40/s1, Figure S1: Deletion of exon10 induces a protein processing defect in D1152H-CFTR expressed in HEK293 cells. Figure S2: Individual lung function (FEV1pp) measurements from D1152H/D1152H patient over time.
