# Supplementary material for: The CFTR Mutation c.3453G > C (D1152H) Confers an Anion Selectivity Defect in Primary Airway Tissue that Can be Rescued by Ivacaftor
Source: J Pers Med. 2020 May 13;10(2):40. doi: 10.3390/jpm10020040 (PMC7354675; doi:10.3390/jpm10020040)
Supplement: Supplementary file 1 [file jpm-10-00040-s001.pdf]

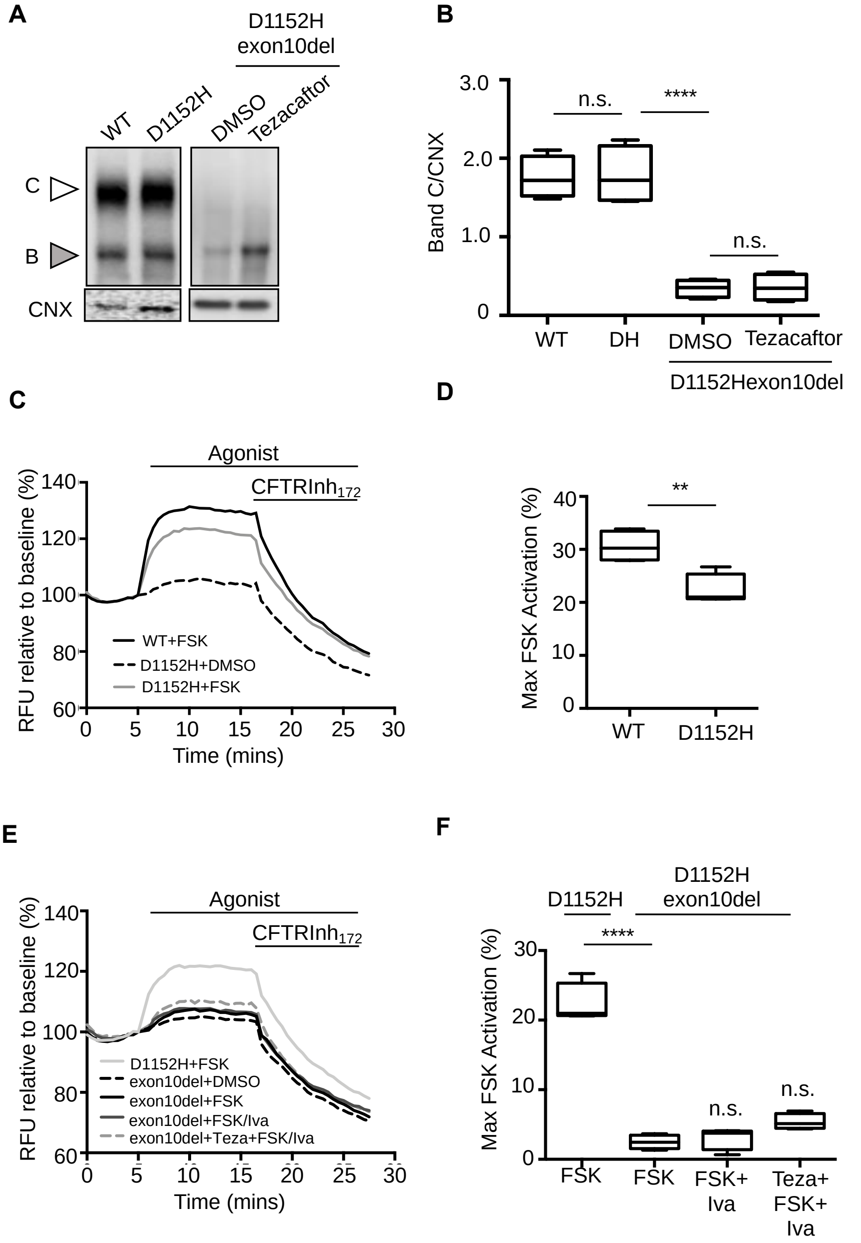

Figure S1. Deletion of exon10 induces a protein processing defect in D1152H-CFTR expressed in HEK293 cells. **(A)** HEK293 cells were transiently transfected with WT-, D1152H- or exon10del/D1152H-CFTR and pre-treated with DMSO or tezacaftor (1 μM) for 24h at 37 °C. The CFTR constructs were detected with an antibody against the C-terminus of CFTR (mAb 596). C, mature complex-glycosylated CFTR; B, immature core-glycosylated CFTR. (**B**) Bars represent the mean (±SD) of the ratio C/(C + B) from five independent experiments. There was a statistical difference between D1152H and D1152H exon10del (**** $p < 0.0001$) but not between DMSO and tezacaftor treated D1152H/exon10del HEK293 cells. (**C**) Representative trace of FLIPR assay in HEK293 cells expressing WT- and D1152H-CFTR at 37 °C. (**D**) Bar graph shows the summary (mean ± SD) of the maximal response to FSK activation from four independent experiments (four technical replicates each experiment) (** $p < 0.01$). (**E**) Representative trace of chloride efflux in D1152H-CFTR and exon10del/ D1152H-CFTR using the fluorometric imaging plate reader membrane depolarization assay in HEK293 cells at 37 °C. Following 5 min baseline measurement, 10μM FSK +/- 1 μM ivacaftor was added. After 10 min incubation, CFTR inhibitor (CFTRinh-172, 10 μM) was added to deactivate CFTR. (**F**) Bar graph shows the mean (±SD) of the maximal FSK activation from four independent experiments. There was a statistical difference between D1152H and D1152H exon10del (**** $p < 0.0001$) but not between FSK and FSK+ivacaftor (VX-770) or FSK+tezacaftor+ivacator treated D1152H/exon10del HEK293 cells.

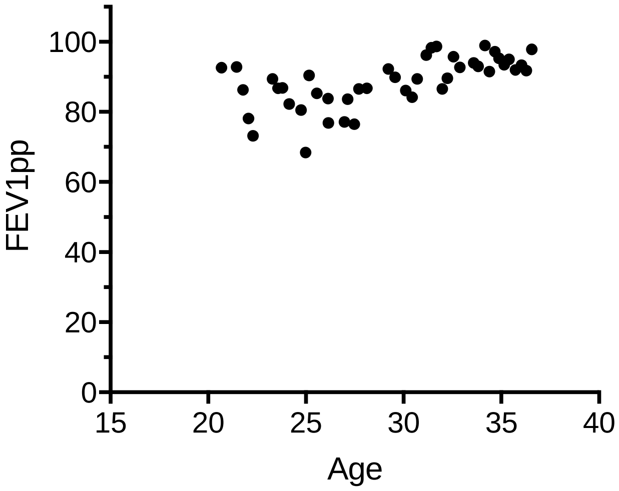

Figure S2. Individual lung function (FEV1pp) measurements from D1152H/D1152H patient over time. The graph shows the lung function measure FEV1pp, which measures the forced expiratory volume in one sec as percent predicted, of the D1152H homozygous patient over a period of 17 years. In contrast to classic CF patients there is no decline of this parameter over time.
